# Single-Incision Percutaneous Closure of Pediatric Inguinal Hernia: A New Modification for Intracorporeal Suture Knotting

**DOI:** 10.1155/2020/5610513

**Published:** 2020-08-06

**Authors:** Ahmed Abdelghaffar Helal, Mohammad Daboos, Alsayed Othman, Muhammad Abdelhafez

**Affiliations:** ^1^Pediatric Surgery Department, Al-Azhar University, Cairo, Egypt; ^2^Al-Houssain University Hospital, Darrasa, Cairo, Egypt

## Abstract

**Background:**

Single-incision percutaneous closure (SIPC) of pediatric inguinal hernia under laparoscopic guidance is a well-developed feasible technique; however, suture knotting remains a major challenge during this technique. Most laparoscopic surgeons prefer extracorporeal subcutaneous suture knotting, which may be associated with consequent formation of stitch sinus and increased recurrence rate. On the other hand, intracorporeal suture knotting necessitates the availability of special devices or homemade instruments with a long learning curve. Therefore, the present study innovates new and simple modification allowing intracorporeal suture knotting during SIPC of pediatric inguinal hernia that does not require any special operating devices or homemade instruments. *Patients and Methods*. Four-hundred children suffering from inguinal hernia of congenital type, submitted to SIPC using Epidural needle (EN), under laparoscopic guidance with intracorporeal suture knotting.

**Results:**

Children ages were 6 months to 10 years (the range). There were 300 boys and 100 girls, and two-hundred children suffered from left side hernia, 150 with right-side hernia, and 50 children with both left- and right-side hernia. 10 ± 2.2 minutes was the recorded operation time in one side hernia repair, while 14 ± 4.3 minutes was recorded for both side repair. Postoperative results reported recurrent hernia in one child and postoperative hydrocele in 3 children which resolved spontaneously after 3 weeks of follow-up.

**Conclusion:**

Intracorporeal suture knotting during SIPC of pediatric inguinal hernia allows for the transformation of a formally extraperitoneal procedure to an intraperitoneal procedure. This new modification for intracorporeal suture knotting does not require any special operating devices or homemade instruments. It seems to be an attractive way during SIPC of pediatric inguinal hernia when intracorporeal suture knotting is considered.

## 1. Introduction

Single-incision percutaneous closure (SIPC) of opened internal inguinal ring (IIR), under laparoscopic guidance, is an excellent technique [1, 2]. Most of the published reports were concerned with rapid and simple extracorporeal suture repair, where the knot was buried subcutaneously, as intracorporeal suture knotting necessitates the availability of special devices or homemade instruments with a long learning curve. However, this subcutaneous knot burring may be associated with some drawbacks as prickle sensation over the stitch site or development of stitch sinus (with subsequent abscess or granuloma formation), if the knot was not buried adequately, especially in thin and small children [3, 4]. Moreover, the suture knotting above the abdominal wall muscles may later become lose with possible hernia recurrence. Additionally, inclusion of some subcutaneous nerves within the knot may occur causing unexplained postoperative paresthesia or pain [5–10]. Herein, the present study innovates new and simple modification allowing intracorporeal suture knotting during SIPC of pediatric inguinal hernia that does not require any special operating devices or homemade instruments.

## 2. Patients and Methods

The present study was prospective and carried out at the Pediatric Surgery Department, Al-Azhar University Hospitals, Cairo, Egypt, from Jan 2016 to May 2019. Children with unilateral or bilateral congenital inguinal hernia were enrolled in this study. There was no age or sex limitation. Recurrent hernia and hernia with patulous IIR (>2 cm) or need muscular arch repair were excluded from this study. 450 hernias were repaired using single-incision percutaneous closure (SIPC) under laparoscopic guidance with intracorporeal suture knotting. Complete history, full physical examination, usual preoperative investigations, and Doppler examination of both tests were done for all cases. Primary outcome measurements were operation time, operative mishaps, and technical feasibility. Secondary outcome assessments were hernia recurrence and testicular atrophy. All cases were operated by the same team, and technical details were the same for all patients. Ethical committee approval was obtained, and all parents signed a written informed consent.

## 3. Operative Details

**Figure 1:**
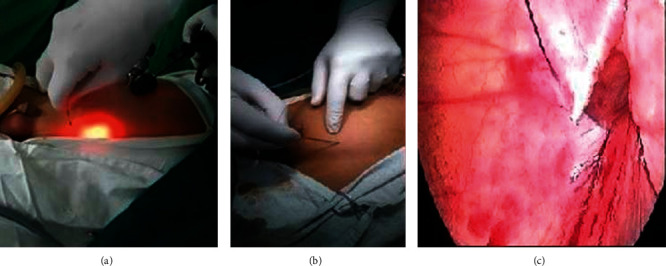
(a, b) Percutaneous insertion of EN containing 3/0 prolene suture through anterior abdominal wall; (c) EN manipulated extraperitoneally to close the IIR.

**Figure 2:**
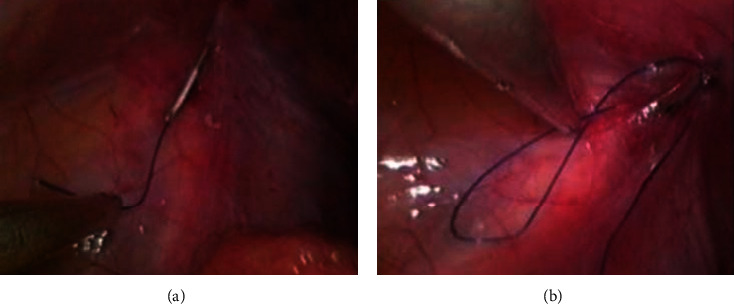
(a, b) Thread ends were grasped from EN and pulled outside the abdomen.

**Figure 3:**
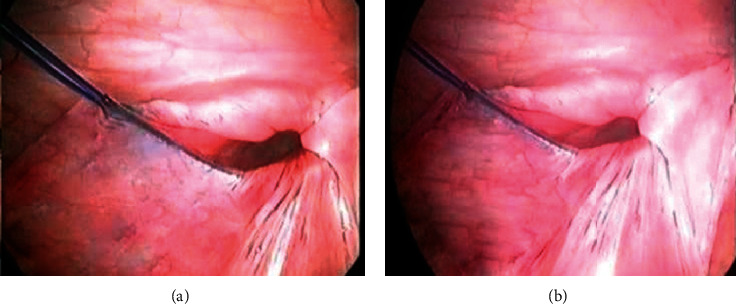
(a, b) Complete encirclement of IIR with both ends at 9 O'clock meridian around IIR.

**Figure 4:**
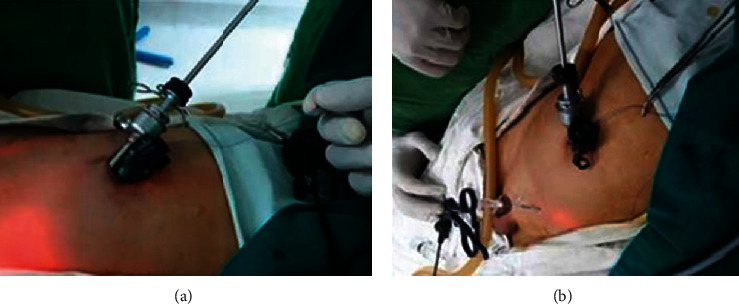
(a, b) Thread ends were passed through transumbilical 3 mm port and held with mosquito forceps.

**Figure 5:**
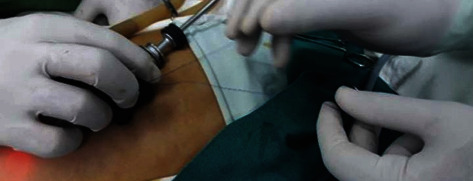
Thread ends were passing outside the umbilicus, and the self-sliding extracorporeal clinch knot was tied.

**Figure 6:**
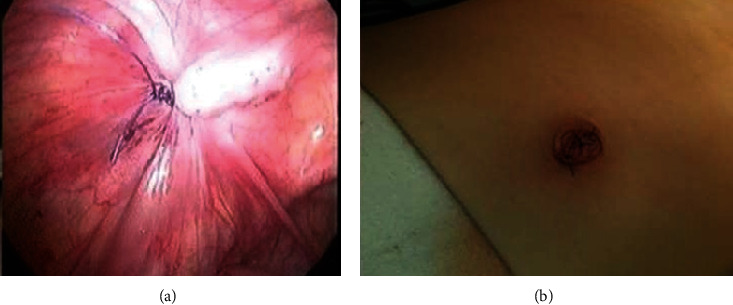
(a) At the end of the repair with intracorporeal suture knotting; (b) after umbilical closure.

General endotracheal intubation was given, and the child was placed supine. A longitudinal transumbilical incision was done, 5 mm telescope was inserted in the center of umbilical cicatrix, and then 3 mm laparoscopic port was introduced few millimeters apart through separate fascial entry. Pneumoperitoneum was created at 8 to 12 mmHg (depends on age). Pelvis and both internal inguinal rings were inspected and evaluated for feasibility of the technique (the technique was applied only for internal inguinal ring with a diameter of 2 cm or less which was equal to distance between maximally opened jaws of 3 mm laparoscopic Maryland; patulous internal inguinal ring needs muscular repair and it is excluded from the present study). Epidural needle gauge-18 (EN) was introduced 2 cm superior-lateral to the right IIR and 2 cm superior-medial to the left IIR (Figure 1), till the tip of the needle appeared just extraperitoneal, and then 7–10 ml saline was injected through EN for hydrodissection. The peritoneum was stretched in the front of EN by transumbilical Maryland, then EN was manipulated extraperitoneal, starting at 3 O'clock position of IIR on the RT side, and it is manipulated on the inferior edge of IIR to penetrate the peritoneal cavity at 9 O'clock (RT side). The thread was introduced from the outside through EN and picked up from it by 3 mm Maryland and then passed out through the transumbilical port. After that, EN was reintroduced at 3 O'clock, and it was manipulated on superior edge of IIR extraperitoneal to pass through pervious peritoneal puncture at 9 O'clock, where the second end of the thread was picked and pulled to the outside using transumbilical Maryland (Figures 2–4) (the steps are the mirror image on the LT side). After that, the knot tying was performed using extracorporeal self-sliding clinch knot (as demonstrated by Weston[11]), followed by intracorporeal suture knotting, and then both ends of the thread were cut 2 cm away from the knot. At the end, the umbilicus was closed using absorbable 3/o suture (Figures 5 and 6).

## 4. Results

Four-hundred fifty inguinal hernias were repaired using single-incision percutaneous closure under laparoscopic guidance with intracorporeal suture knotting in all cases. They were 300 boys and 100 girls. Contralateral patent process vaginalis was discovered laparoscopically in 50 children, and they closed at the same operation. The mean age was 6 ± 2.2 years (age range from 6 months to 10 years).  Table 1 shows demographic characteristics of patients. Mean operation time was 10 ± 2.3 minutes for single-side hernia and 14 ± 4.3 minutes for both side hernias. Postoperative follow-up was 12 months (mean) (ranging from 6 to 24 months). A single child with recurrent hernia was reported, and 3 cases with hydrocele formation which disappeared conservatively after 3 weeks. Atrophy of the testes was not reported postoperatively in any child (clinically and by follow-up testicular doppler). All operations succeeded laparoscopically. All children went to their home 8 to 10 hours postoperatively.

## 5. Discussion

Laparoscopic repair of inguinal hernia in children was started with multiport technique. After that, Helal described the laparoscopic single instrument technique. At the moment, single-port and single-incision laparoscopic repair of inguinal hernia has been confirmed. In 2015, Lee et al. demonstrated that using laparoscopic purse-string suture around open IIR is enough for pediatric inguinal hernia repair [12–14].

**Figure 7:**
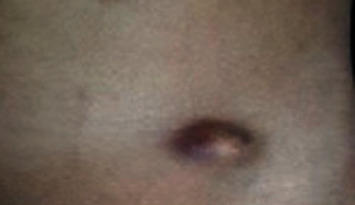
Subcutaneous abscess following laparoscopic-assisted extraperitoneal closure of inguinal hernia with extracorporeal suture tying and subcutaneous knotting.

Recently, single-incision percutaneous closure of pediatric inguinal hernia under laparoscopic guidance was described by many pediatric surgeons, as a straightforward procedure with rapid extracorporeal suture knotting. However, this subcutaneous suture knotting was reported to cause some unavoidable mishaps, such as stitch sinus with subsequent abscess or granuloma formation if the knot was not buried adequately (Figure 7). Moreover, muscle entrapment within the suture may increase the incidence of hernia recurrence. Also, inclusions of subcutaneous tissues within the suture may cause postoperative paresthesia or inguinal pain [15–19]. On the other hand, intracorporeal suture knotting during SIPC is challenging, due to bad ergonomics caused by limited access and movement space that required higher experience, certain skills level, long learning curve, and undutiful and it is a time consuming step [20–24].

In the present study, we applied the same principles of Patkowski technique [25] for needlescopic-assisted repair of inguinal hernia. However, unlike Patkowski technique, which uses only one umbilical port, his knot was tightened extracorporeally and buried subcutaneously. Herein, we succeeded to apply intracorporeal self-sliding locking knot through second transumbilical 3 mm port, where the whole possible drawbacks of subcutaneous knotting were totally eliminated. Moreover, the presence of 3 mm transumbilical working instrument allowed stretching the peritoneum in front of EN and facilitates the retrieval of both thread ends and knot sliding. The present technique describes for the first time the use of self-sliding clinch knot (as demonstrated by Weston [11]) during SIPC of pediatric inguinal hernia [26, 27]. The clinch knot is kind of slip and self-sliding knot; it does not require tie pusher instrument. It is used for securing a fishing lure, hook, or swivel to a fishing line by fishermen and has historically proven to be secured. In addition, it takes less time to tie and it is not as bulky as other knots. It has been used as an alternative to the Endoloop and as the preferred knot to secure the Endo-Knot. Additionally, the clinch knot preserves the tactile sensation of the laparoscopic surgeon and allows for optimum adjustments of the strength applied over tissues. There was no age or sex limitation to the present technique. However, the main concern for the technique was the IIR diameter, as the technique was applied only for IIR with a diameter of 2 cm or less which was measured by the distance between maximally opened jaws of 3 mm laparoscopic Maryland; wider IIR may needs muscular repair and it is excluded from the present study [11, 24–28].

Another potential advantage of the present technique is in managing some cases of incarcerated hernias, as the presence of transumbilical instrument can be carefully used to pull the incarcerated content back, with the assistance of external manual pressure; after that, closure of the hernia defect can be done as usual. In our experience, in two incarcerated hernias in 4 and 7 years old boys (both cases not included in the present study), the content was reduced by the help of transumbilical Maryland, and hernia repair was completed laparoscopically without the need for conversion or additional instrument placement.

Some authors question the value of intracorporeal suture knotting in SIPC of pediatric inguinal hernia and may consider it a sort of overdoing. To answer this question, it may be more appropriate to compare the repaired intracorporeal cohort with an extracorporeal cohort as well as comparing percutaneous closure technique with other transperitoneal closure techniques. However, in this preliminary report, we tried to address this issue from a different perspective by evaluating percutaneous closure with this new modification for intracorporeal suture knotting and for the first time use of self-sliding clinch knot during SIPC of pediatric inguinal hernia, regarding operative time, feasibility of the technique, and complications as hernia recurrence, postoperative hydrocele formation, and testicular atrophy after the repair. Finally, future prospective controlled randomized studies comparing SIPC of pediatric inguinal hernia with intracorporeal suture knotting group and extracorporeal suture knotting group is recommended to evaluate the superiority of one technique.

## 6. Conclusion

Intracorporeal suture knotting during SIPC of pediatric inguinal hernia allows for the transformation of a formally extraperitoneal procedure to an intraperitoneal procedure. This new modification for intracorporeal suture knotting does not require any special operating devices or homemade instruments. It seems to be an attractive way during SIPC of inguinal hernia in children when intracorporeal suture knotting is considered.

## Figures and Tables

**Table 1:** Demographic data of patients.

Demographic data	Number	Percent (%)
Number of patients	400	100
Sex	300 males and 100 females	
Age Mean range	6 ± 2.2 years 6 months and 10 years	

*Presentation*		
Clinical	Number	Percent (%)
Left inguinal hernia Right inguinal hernia	200 150	50 37.5
Operative	Number	Percent (%)
Bilateral inguinal hernia	50 (clinically left)	12.5

*Complications*		
Type	Number	Percent (%)
Intraoperative	Nil	0
Postoperative	1 case of hernia recurrence 3 cases of postoperative hydrocele	0.25 0.75

## Data Availability

Data are not available to other researchers because they are from a registry or institutional database of patients providing routinely collected data.
